# Modelling searching movements of the front leg in the stick insect by means of a neuro-muscular model

**DOI:** 10.1186/1471-2202-16-S1-P50

**Published:** 2015-12-18

**Authors:** Tibor I Toth, Eva Berg, Ansgar Büschges, Joachim Schmidt, Silvia Daun-Gruhn

**Affiliations:** 1 Heisenberg Research Group of Computational Biology, Institute of Zoology, University of Cologne, 50674 Cologne, Germany; 2 Department of Animal Physiology, Institute of Zoology, University of Cologne, 50674 Cologne, Germany

## 

Searching movements, beside locomotion, are, perhaps, the most important motor activities of the front legs in stick insects. In a recent study [1], the kinematics of these movements were thoroughly investigated. In these experiments the animal was restricted such that its front leg could freely move in the plane perpendicular to the longitudinal axis of the animal, only. Having induced leg movements in the stick insect, an obstacle was put in the way of the distal tibia at different angle positions with respect to the horizontal axis. The obstacle was however removed immediately after collision with the insect's leg. Leg movements before and after the animal's touching of the obstacle were recorded and analyzed. In particular, the angle between the horizontal axis and the femur (β), and that between the femur and tibia (γ) were measured. In addition, the angle (α) describing the position of the distal tibia was also determined. It was found that the front leg after collision with the obstacle showed modified searching movements that could be regarded as targeted response expressed by the time courses of the angles α, β and γ. The specific properties of the targeted searching movements depended on the position of the obstacle.

Using our existing model that was constructed to emulate stepping of a single middle leg [2], we tried to mimic the aforementioned experiments. First, we replaced the anatomical data of the middle leg with those of the front leg of the stick insect. We then fixed the protractor-retractor angle at 90° in the model, as it was done in the experiments. We implemented the searching movements by appropriate activation or deactivation of fast and slow muscle groups of the levator-depressor and the extensor-flexor neuro-muscular system. At touch of an obstacle, all muscle activity was stopped for a very short time, and the recruitment of the muscles in the individual muscle groups was also reduced to a minimum. Restoring the activity and recruitment in the individual muscle group took place depending on the position of the obstacle. We thus succeeded in replicating the main properties of the leg movements before and after its touching the obstacle. We found that the time course of the recruitment of the fibres in the fast levator and depressor muscles crucially affected the time courses of the angles α, β and γ, hence the overall (2-dimensional) movement of the leg. The threshold value of the angle β at which the extension switches to flexion and vice versa in the extensor-flexor neuro-muscular system has turned out to be another important system parameter. At lowering the value of this parameter, the time course of γ becomes increasingly erratic, and the tibia eventually stops oscillating. Such events could also be observed in the experiments.

The good agreement between experimental and simulation results suggests that the properties and parameters used in the model to mimic search behaviour, might also be those that affect the searching movements in the stick insect in an analogous way.
